# Changes in health communication in the age of COVID-19: A study on the dissemination of preprints to the public

**DOI:** 10.3389/fpubh.2023.1078115

**Published:** 2023-02-08

**Authors:** Li Zeng

**Affiliations:** Department of Journalism and Communication, School of Information Technology in Education, South China Normal University, Guangzhou, China

**Keywords:** science communication, preprints, COVID-19, scientific journalism, open science, health communication

## Abstract

**Introduction:**

Preprints have become an important tool for meeting the challenges of health communication in the context of COVID-19. They allow scientists to disseminate their results more quickly due to the absence of a peer review process. Preprints have been well-received by scientists, however, there have been concerns about the exposure of wider public audiences to preprints due in part to this lack of peer review.

**Methods:**

The aim of this study is to examine the dissemination of preprints on medRxiv and bioRxiv during the COVID-19 pandemic using content analysis and statistical analysis.

**Results:**

Our findings show that preprints have played an unprecedented role in disseminating COVID-19-related science results to the public.

**Discussion:**

While the overall media coverage of preprints is unsatisfactory, digital native news media performed better than legacy media in reporting preprints, which means that we could make the most of digital native media to improve health communication. This study contributes to understanding how science communication has evolved in response to the COVID-19 pandemic and provides some practical recommendations.

## 1. Introduction

Science communication faces opportunities and challenges in the context of COVID-19. There has been an increase in public interest in scientific research during the pandemic (1, 2). These needs, however, cannot be met by traditional models of science communication. Science communication used to involving scientists publishing their findings in peer-reviewed scholarly journals and then distributing them to the general public through journalistic reporting. While the publication process is slow for scholarly journals, peer-reviewed journals in the fields of public health and medicine, for example, have a publication cycle of ~3 months (3). Publication lag times became a serious problem during the pandemic. According to one study, SARS ended before 93% of the SARS studies were published (4).

Preprint platforms are a new means of disseminating scientific research. They provide rapid dissemination, citation advantage, receiving feedback, and so on. Therefore, they are regarded as an accelerator of scholarly communication (5–7). Preprints are becoming increasingly popular in various disciplines, such as life sciences (8), mathematics (6), and information sciences (9).

Preprints have become an important tool in meeting these challenges. Researchers upload them on the preprint platform without any peer review. Preprints are usually published before the formal publication process. They are an important scholarly communication tool during the pandemic because of their rapid publication speed and that they are open access (10–12).

Preprints were generally well-received by scientists, but there are concerns about their use for public scientific communication. Preprints can be an efficient means of delivering information to the public. Meanwhile, inappropriate dissemination of preprints can have serious consequences (13). As preprints are not peer-reviewed, quality problems can arise, causing confusion and panic among the public. A paper published on bioRxiv suggested that the COVID-19 virus was genetically engineered because it is similar to HIV. It received widespread public attention, with some citing it as evidence that COVID-19 is a biological weapon (12, 14)^1^.

In this context, we need to examine the role of preprints in disseminating COVID-19 research to the public.

## 2. Literature review

Scientific research results are increasingly being covered in the mass media (15). Both science news and non-science news are increasingly reporting papers from scholarly journals (16, 17). Over 99% of scientific papers covered by mass media are related to health and medicine (18–20).

There are two main perspectives on the mass media's coverage of scientific papers. One is from a bibliometrics perspective, examining whether mass media distribution can increase the number of citations of the papers (21, 22). The other concern is the accuracy of reporting. Researchers have questioned the reliability of news coverage of academic content in the mass media, stating that between half and 90% of news coverage has problems (23, 24). These problems include omitting contextual information (15, 25), exaggerated conclusions (26), and misleading causal claims (27), which are widespread across a wide range of fields (28, 29).

COVID-19 has led to an unprecedented use of preprints (12, 30). They are seen to accelerate the dissemination of scientific discoveries in response to infectious disease outbreaks (31). Preprints related to COVID-19 have increased more than 100-fold compared to research on other viruses (32).

In addition, COVID-19 prompts the dissemination of preprints beyond the academic community to the wider public (13). Many mainstream journalists have to report on preprints due to the pandemic, which has even become a frequent practice (33–35). While the role of preprints was well-recognized within the academic community, their dissemination to the public raised concerns. Journalists often consider preprints that have not been peer-reviewed as reliable sources of information, and the reporting process follows that of peer-reviewed articles (34, 36). Since it is difficult for non-scientists to distinguish between preprints and peer-reviewed papers, this can lead to confusion and distortion, ultimately resulting in fake news, conspiracy theories, and extremist ideologies (13, 37–39).

Studies focusing on reporting preprints in mass media within the context of the pandemic can be divided into two categories. First, a qualitative approach focuses primarily on experience, summarizing the news reporting practices of preprints by journalists. They explain why preprint coverage has become so extensive and the problems associated with the reporting practice (33, 35, 40).

Second, empirical studies are conducted. Fleerackers et al. examine the reporting of preprints in 15 media outlets (41). Some studies examine preprint coverage across specialized regions, such as the United States, the United Kingdom, Brazil, and South Africa. These studies provide an analytical frame for preprint coverage and indicate the proportion of preprints that are not correctly reported, ranging from 27.6 to 59% (42–44).

Together these studies provide important insights into how preprints are communicated to the public. This paper expands on existing research in four ways. First, an empirical study is conducted to investigate whether and how the pandemic has led to wider dissemination of preprints. Secondly, the study sample was selected based on the preprints published on preprint platforms that failed to appear in peer-reviewed journals. Some preprint papers are eventually published in peer-reviewed journals, while previous studies have often failed to make this distinction, and such confusion can affect the accuracy of conclusions. This distinction is important because papers that do not undergo peer review are more likely to have quality issues, and the potential consequences of inaccurate reporting of these papers could be severe. As a result, this study will focus on preprints that do not ultimately appear in peer-reviewed journals. Thirdly, this paper will explore what factors influence the inaccurate reporting of preprints. Lastly, it examines data from social media compared to the mass media.

In this paper, we address the following research questions:

RQ1. How does the COVID-19 pandemic influence the breadth of the audience exposed to preprints?
RQ2. How have COVID-19-related preprints been covered in the news media?
RQ3. What factors influence the accuracy of the media coverage of the preprints?

## 3. Methods

### 3.1. Sample selection

The sample for this study was selected from preprints published in medRxiv and bioRxiv during the early stage of the pandemic. I chose these two platforms because they are the most important preprint platforms for biological and medical research (45, 46). The early stage of the pandemic is selected because the lack of information at this time offers opportunities for widespread dissemination of preprint papers (12). Considering that the timing of the early stage of the pandemic varies from country to country (47–50), we chose preprints published from 1 January to 30 June 2020, which provides an overview of the first wave of the pandemic for most countries.

We use the dataset from Fraser et al. as the data source (51). This dataset contains information on all papers published on the preprint platforms medRxiv and bioRxiv from 1 January to 31 October 2020, along with their metadata (e.g., title, doi, author information, etc.) as well as whether the preprint relates to COVID-19. Furthermore, the publication status of the paper was marked, making the dataset ideal for this study^2^.

I extracted from the dataset 26,425 preprints published on the bioRxiv and medRxiv platforms from 1 January to 31 June 2020^3^. After removing the 8,899 (33.7%) papers that had already been published in peer-reviewed journals, 17,526 (66.3%) papers remained. Of these, 4,269 (16.2%) were COVID-19 related, and 13,257 (50.2%) were non-COVID-19 papers. And bioRxiv contributed 841 papers and medRxiv contributed 3,428 papers. Additionally, all preprints from bioRxiv and medRxiv from 2013 to the end of 2019 were obtained as comparative data, excluding articles that had already been published in peer-reviewed journals.

### 3.2. Data collection

Altmetric, a service that tracks the public attention research papers receive, was used to collect data on disseminating preprint papers in the public domain (52). I queried the DOI of each preprint in our sample using the Altmetrics API and accessed the records of mentions in news media and Twitter activity for each preprint.

To address research question 2, I selected preprint papers whose news records were ≥3^4^. I did this to identify papers that have the most influence on the public. Approximately 710 papers were selected after filtering. The first news link that was reported on these papers was then retrieved. Finally, LexisNexis databases and the Internet are used for extracting the content of these news stories by crawlers and manual crawling^5^. After removing 190 items of non-English content, 38 items of original deleted content, and 14 items of content that required payment but were not in the LexisNexis database, 468 items remained and were used as a sample for further content analysis.

### 3.3. Content analysis

This study developed a codebook based on the research of Fleerackers et al. (41) and van Schalkwyk and Dudek (42). In the codebook, three frames were used to determine whether the news media accurately reported the preprints. These included using the term “preprint,” the declaration that the article was not peer-reviewed, and the statement that it was preliminary research. As these frames were relatively well-identified, we searched for keywords mentioned as coding methods. Then 10% of the articles were randomly selected and manually checked to refine the keywords, and “preprint,” “preprints,” “peer-reviewed,” “early,” etc., were added as search keywords. The codebook is shown below. Table 1 presents the description, source, and examples of each code.

**Table 1:** Overview of codes.

**Code**	**Description**	**References**	**Examples**
Mention preprint	Including “preprint” or “preprints” or “pre-print” or “pre-prints”	Fleerackers et al. (41)	• Their paper is posted online in preprint • In one study, which is available online as a pre-print
Mention not peer reviewed	Search “peer review” or “peer reviewed”	van Schalkwyk and Dudek (42)	• Due to the rapid response nature of the study, it has not yet been peer reviewed or published in a journal • They haven't been through the rigorous peer review process required to publish in scientific journals.
Mention preliminary	Search “preliminary” or “early”	Fleerackers et al. (41)	• Vander Heide, from LSU, who reported preliminary findings on 10 patients • Early research posted on Monday to the online health sciences server medRxiv found a nearly 18% drop

For words with multiple meanings, such as “preliminary” and “early,” it was confirmed from the text manually.

To address RQ3, the platforms which report the preprints were categorized in two ways, one based on media content and the other based on the media type. This study categorized the media content as medical publications, business publications, biology publications, science publications and general News publications. As for the media type, they are divided into legacy media, news aggregators and digital-native news platforms.

Tables 2, 3 show the definitions, explanations, and examples of media content and media type.

**Table 2:** Definitions of media content.

**Media content**	**Explanation**	**Example**
Biology publications	Content focused on the biological field	Biospace
Business publications	Content focused on the business field	Business insider
General news publications	Content focused on general news	BBC news
Medical publications	Content focused on the medical field	The medical news
Science publications	Content focused on the scientific field	Scientific American

**Table 3:** Definitions of media type.

**Media type**	**Explanation**	**Example**
Digital-native news platforms	The news media born on the web	Vox.com
Legacy media	Traditional media	Washington post
News aggregators	Websites that aggregate media content	Yahoo! News

### 3.4. Data analysis

The data were analyzed using SPSS 26.0, with the following methods:

(1) Descriptive statistics were applied to the dissemination of preprints before and after COVID-19.
(2) Spearman correlation coefficients were used to examine the relationship between the spread of news and Twitter feeds.
(3) The media type and media content were cross-tabulated with preprint frame adoption scores. The preprint frame adoption scores is the number of pre-printed adoption frames used for each news in the content analysis. Scores ranged from 0 to 3, with higher scores indicating more accurate preprint coverage. These scores were cross-tabulated with the number of frames adopted to understand the differences in the accuracy of preprint reporting across different categories of media.

## 4. Findings

This study aims to examine how the preprints are disseminated to the public in the context of the COVID-19 pandemic. It employs content analysis and statistical analysis to investigate the dissemination of preprints on the preprint platforms medRxiv and bioRxiv. The findings of this study are as follows.

The public's awareness of preprints has increased dramatically in the pandemic, especially for the preprints related to COVID-19. In the following analysis, the preprints published after 2020 are grouped as relevant and irrelevant for the COVID-19 content, as shown in Table 4.

**Table 4:** Altmetric score for COVID-19-related preprints and non-COVID-19-related preprints in news media and Twitter.

	**Content**	***n***	**Min**	**Max**	**Mean**	**Std. Dev**.	**Median**	***P***	**Cohen's *d*-value**
News media	COVID-19	4,269	0	508	4.059	20.499	0	0.000^***^	0.389
	Non-COVID-19	13,257	0	90	0.159	1.171	0		
Twitter	COVID-19	4,269	0	20,799	114.353	758.035	10	0.000^***^	0.266
	Non-COVID-19	13,257	0	1,656	16.221	35.215	7		

^***^P < 0.01.

COVID-19-related preprints are more widely disseminated than non-COVID-19 content, as shown in Table 4. This is evident from the fact that the mean (4.059 vs. 0.159, 114.353 vs. 16.221, respectively) and median (0 vs. 0, 10 vs. 7, respectively) of the COVID-19-related preprints on news and Twitter are both greater than or equal to the non-COVID-19-related preprints. It appears that news media coverage favors COVID-19 preprint content. The medians for COVID-19 and non-COVID-19 in the news media were 0.0/0.0. The test resulted in a *P*-value of 0.000^***^, indicating a significant difference between COVID-19 and non-COVID-19 in the news media. The magnitude of the difference, as measured by Cohen's d, was 0.389, indicating a small difference.

The medians for COVID-19 and non-COVID-19 on Twitter were 7.0/10.0. The test resulted in a *P*-value of 0.000^***^, indicating a significant difference between COVID-19 and non-COVID-19 on Twitter. The magnitude of the difference, as measured by Cohen's d, was 0.266, indicating a small difference. Furthermore, this table suggests that content related to COVID-19 may have larger maximum values. This means that some preprint papers related to COVID-19 may receive a relatively high degree of public exposure.

Table 5 shows that 33% of COVID-19-related preprints were mentioned in the news at least once at an early stage of the epidemic, whereas only 11% of non-COVID-19 preprints were mentioned at least once at the same time. Regarding social media shown Table 5, 99.90% of COVID-19-related preprints received at least two tweet mentions, compared to 95% for non-COVID-19 preprints^6^.

**Table 5:** The proportion of preprints mentioned in news or Twitter.

	**COVID-19-related**	**Non-COVID-19-related**
News	33%	11%
Twitter	99.90%	95%

It shows that the correlation coefficient between news media and TWITTER is 0.538 at a 0.01 level of significance, indicating a significant positive correlation between news and TWITTER. The result suggests a strong similarity in the perspective of public attention.

While on the other hand, the epidemic has a greater impact on the dissemination of preprints in the news media than on social media. The change in preprint distribution in the news media is greater than the change in social media after the epidemic. In addition, the difference in the number of COVID-19-related preprints and non-COVID-19 preprints disseminated was also greater in the news media than in social media.

A significant number of preprint papers are not accurately reported by the news media. Half of the news stories fail to mention the preprint frame, which means that readers would regard it as the same as a peer-reviewed paper. And those papers that do mention the preprint frame often lack further explanation.

**Figure 1:**
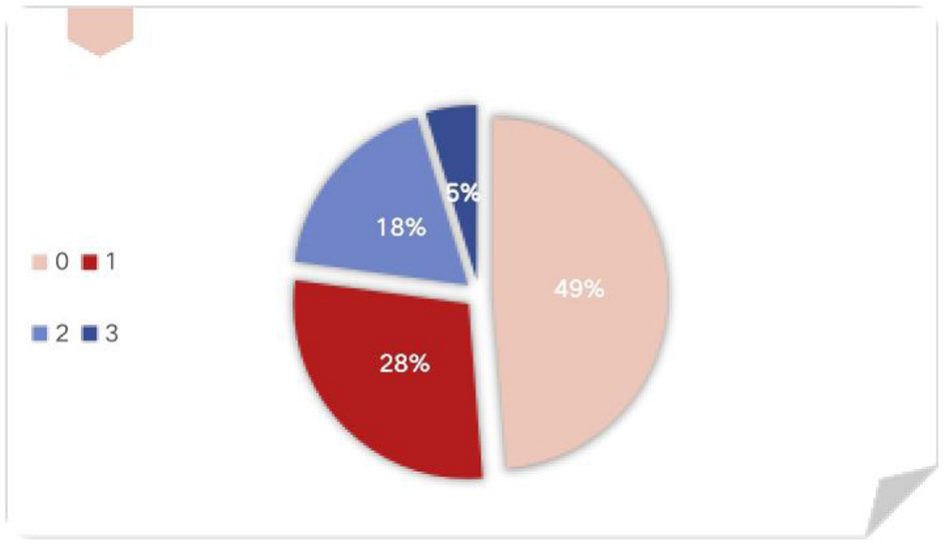
Preprint frame adoption score in the news media.

Figure 1 shows that 49% of the news reports do not mention any preprint frame, which means that readers would regard it as the same as a peer-reviewed paper.

**Figure 2:**
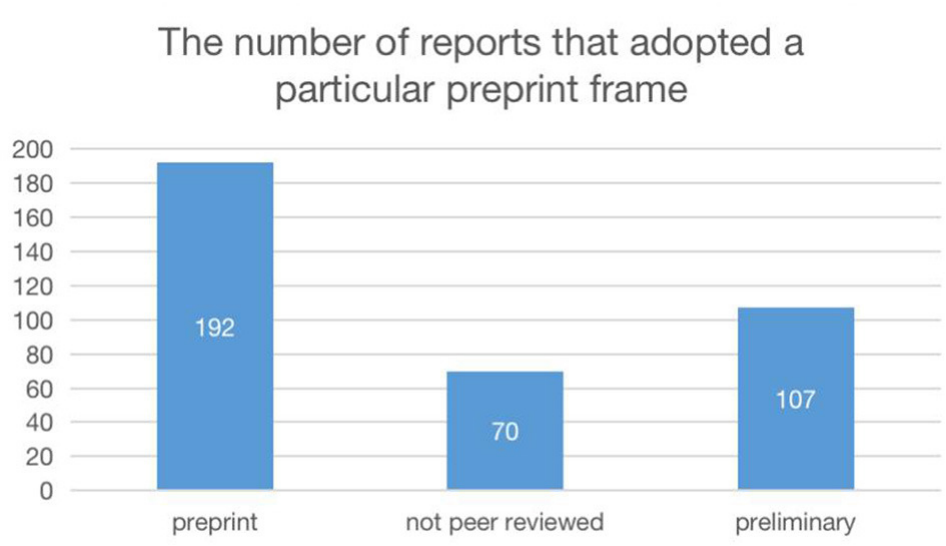
The number of reports that adopted a particular preprint frame.

Figure 2 presents the number of reports that adopted a particular preprint frame. As shown in the figure, the most commonly used approach was mentioning the preprint status of the paper, with 192 (41% of) reports adopting this approach. The second most frequently used approach was mentioning the preliminary nature of the study, with 107 (23% of) reports adopting this approach. Only 70 (15% of) reports mentioned the non-peer-reviewed status of the study. It is clear that the news coverage of the preprint is not accurate.

The media's content characteristics are more likely to influence its coverage of the preprint than the media's type characteristics.

**Figure 3:**
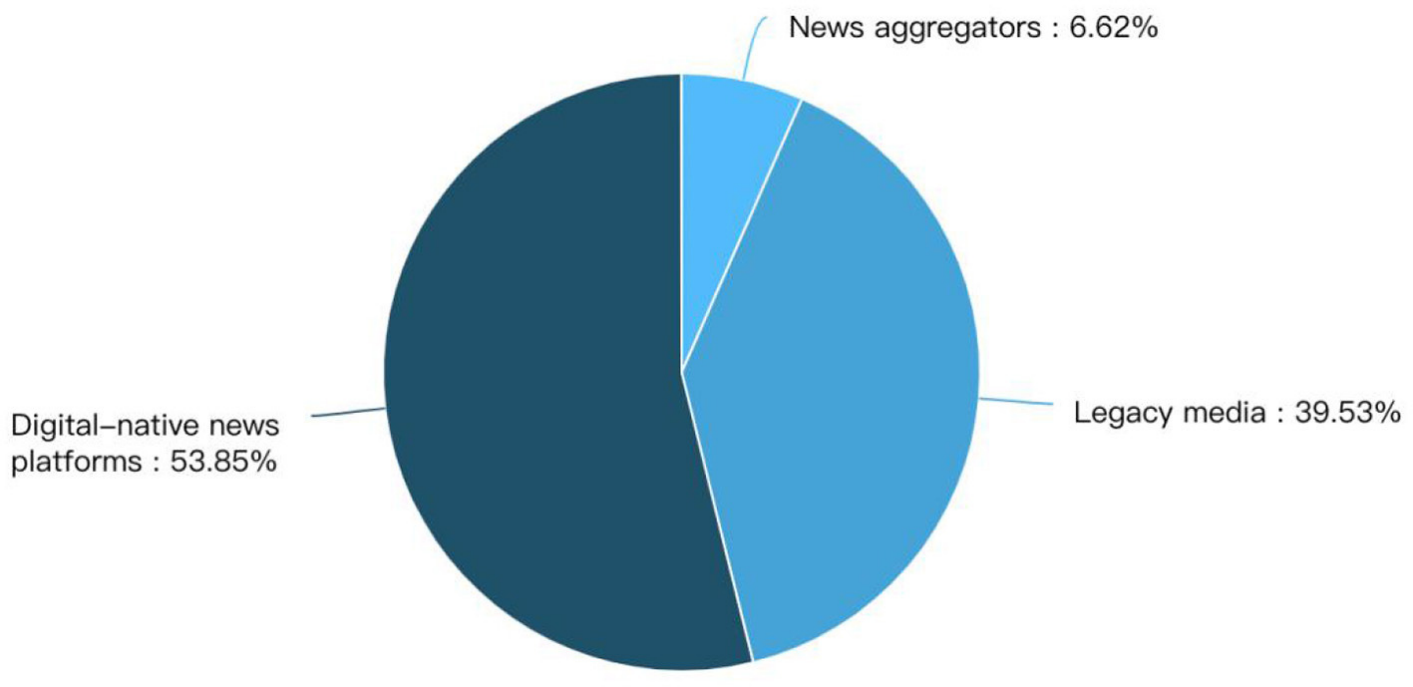
Different media type that cover preprints.

**Figure 4:**
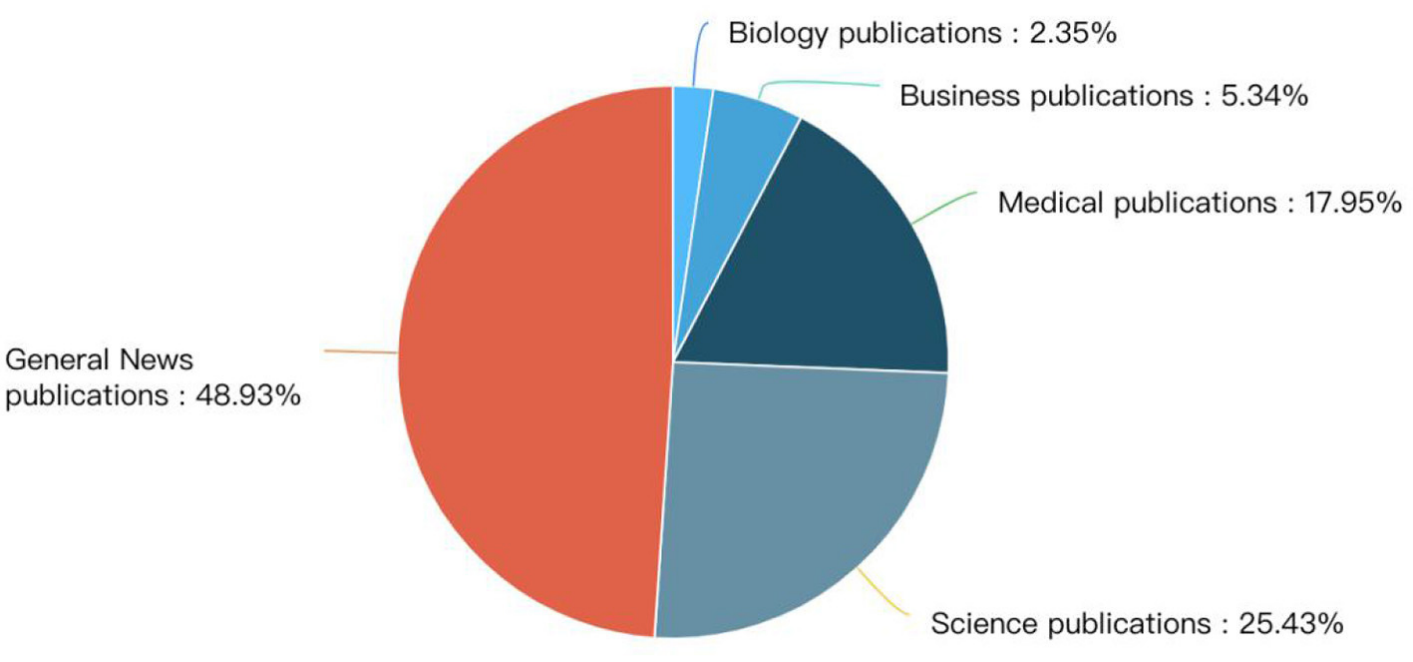
Different media content that cover preprints.

Figures 3, 4 show the media sources. In media type, more than half of the content is published in digital native media and 39% in legacy media. While in terms of media content, the majority is published in general news media.

The results of the chi-square test (cross-tabulation) presented in Table 6 indicate a significant (*p* < 0.05) relationship between media type and preprint frame adoption scores. This suggests that the different media type samples show a difference in preprint frame adoption.

**Table 6:** Chi-square test (cross-tabulation) results for the media type and the number of preprint frames adopter.

	**Media type (%)**					

**Number of preprint frames adopted**	**Digital-native news platforms**	**Legacy media**	**News aggregators**	**Total**	*x* ^2^	***p***
0	104 (41.27)	104 (56.22)	20 (64.52)	228 (48.72)	18.889	0.004^**^
1	74 (29.37)	50 (27.03)	9 (29.03)	133 (28.42)		
2	57 (22.62)	26 (14.05)	2 (6.45)	85 (18.16)		
3	17 (6.75)	5 (2.70)	0 (0.00)	22 (4.70)		
Total	252	185	31	468		

^*^p < 0.05, ^**^p < 0.01.

From the Table above, we can see that using a chi-square test (cross-tabulation) to examine the relationship between media type and preprint frame adoption. The media type shows a 0.01 level of significance for the total score (chi = 18.889, p = 0.004), and the difference in percentage comparison shows that news aggregators selected 0 at 64.52%, which is significantly higher than the average of 48.72%. 56.22% of legacy media selected 0, significantly higher than the average of 48.72%.

**Figure 5:**
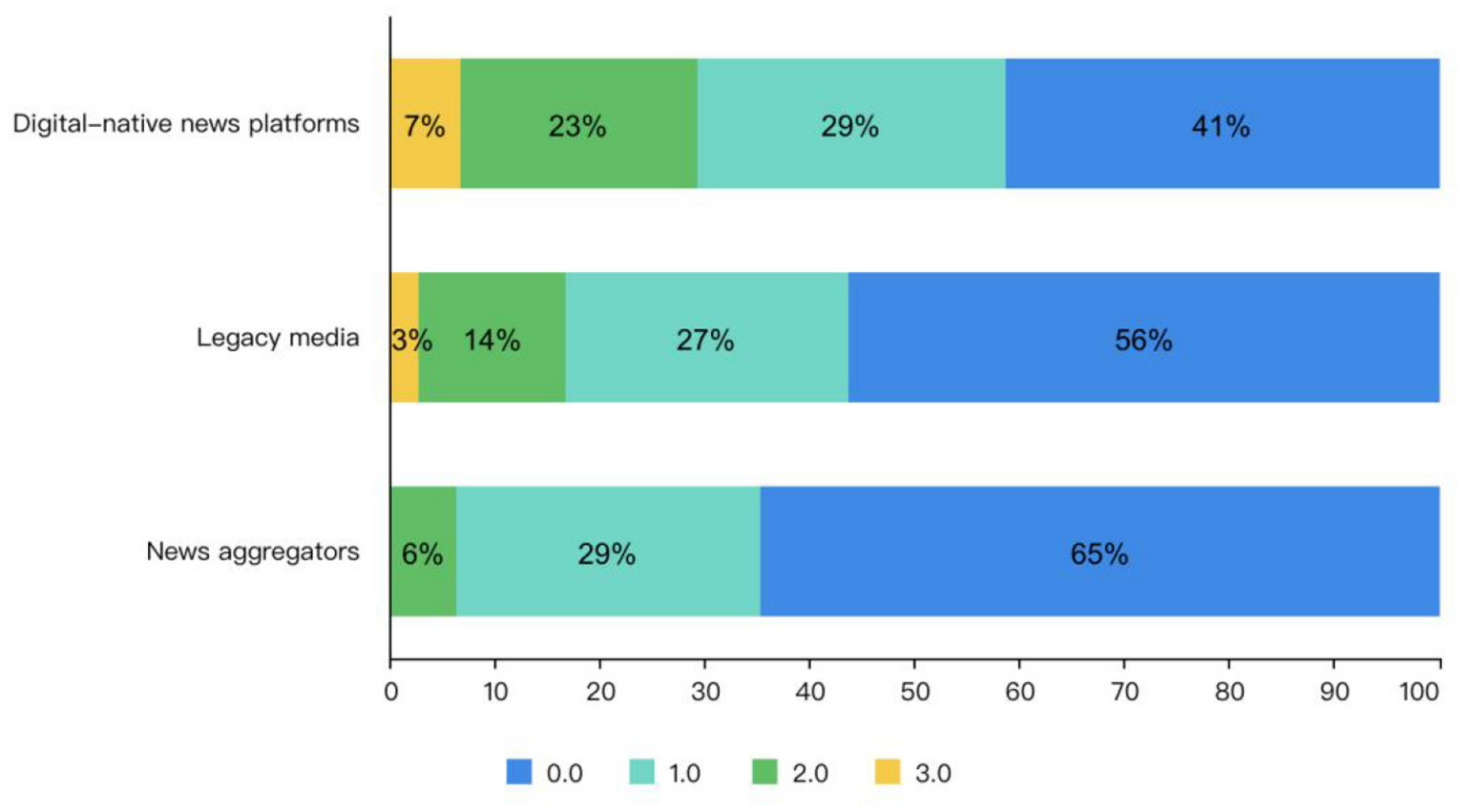
The preprint frame adoption scores of the different media types.

Figure 5 demonstrates the preprint frame adoption scores of the different media types. We can see from the Figure that digital native news platforms have the highest preprint frame adoption scores, with both legacy media and news aggregators below. And this indicates that digital native platforms are more accurate in reporting preprints.

The results of the chi-square test presented in Table 7 indicate a significant (*p* < 0.05) relationship between media content and preprint frame adoption scores. This suggests that the different media content samples show a difference in preprint frame adoption.

**Table 7:** Chi-square test (cross-tabulation) results for the media content and the number of preprint frames adopter.

	**Media content (%)**						
**Number of preprint frames adopted**	**Business publications**	**General news publications**	**Medical/Biology publications**	**Science publications**	**Total**	*x* ^2^	***p***
0	16 (64.00)	131 (57.21)	23 (24.21)	58 (48.74)	228 (48.72)	62.827	0.000^**^
1	7 (28.00)	64 (27.95)	23 (24.21)	39 (32.77)	133 (28.42)		
2	1 (4.00)	26 (11.35)	39 (41.05)	19 (15.97)	85 (18.16)		
3	1 (4.00)	8 (3.49)	10 (10.53)	3 (2.52)	22 (4.70)		
Total	25	229	95	119	468		

^*^p < 0.05, ^**^p < 0.01.

Using a chi-square test (cross-tabulation) to examine the relationship between media content and adoption of preprint frames, the Table above shows that the different media content showed significant adoption of preprint frames (p < 0.05). The media content shows a 0.01 level of significance for the adoption of the preprint frame (chi = 62.827, p = 0.000). To meet the requirements for a chi-square test, we combined the categories of biology and medicine for the purposes of this analysis.

**Figure 6:**
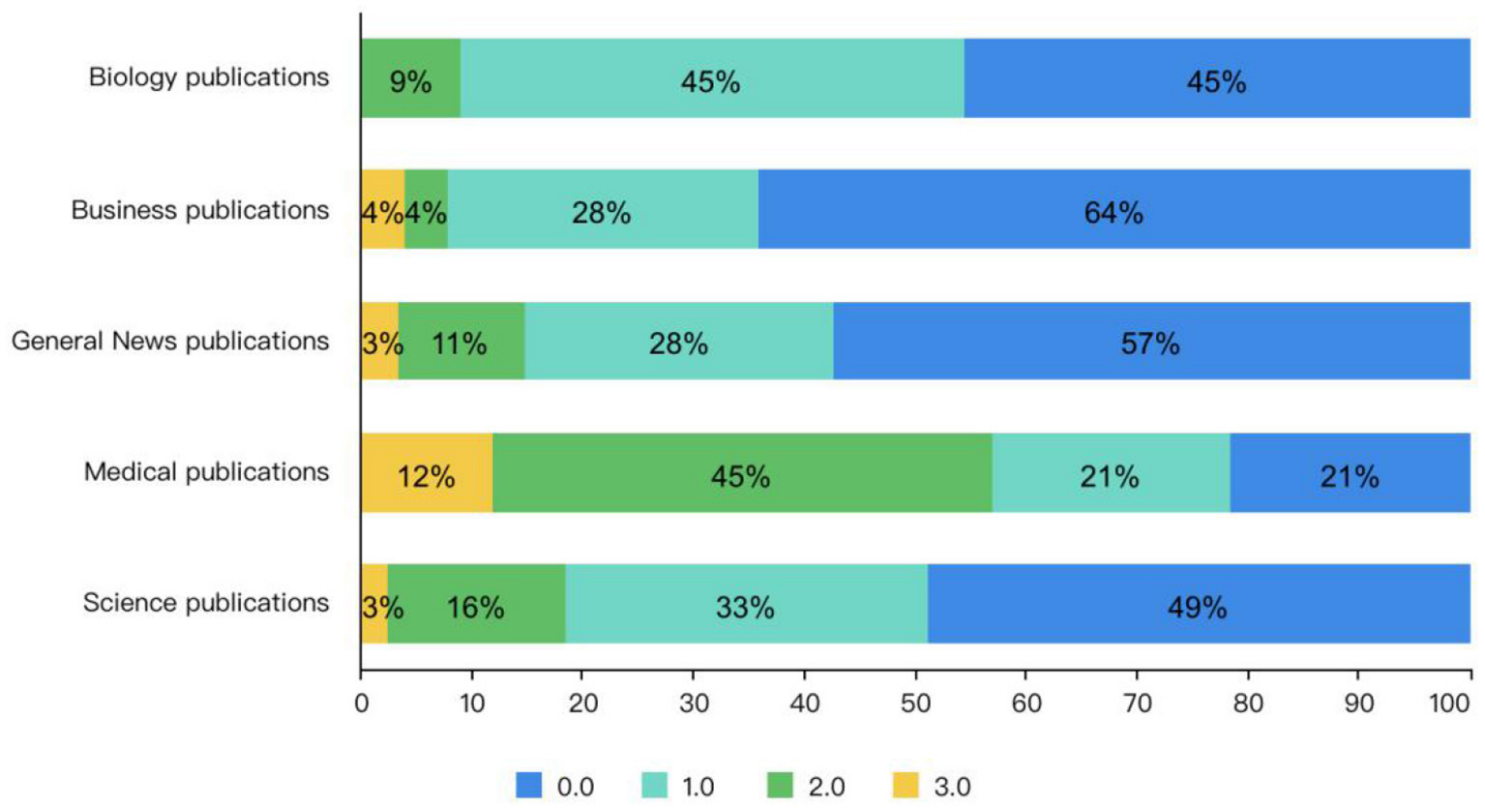
Preprint frame adoption scores of different media content.

Figure 6 illustrates the preprint frame adoption scores of different media content. As can be seen, medical, scientific, and biological categories have scores, which means they report preprints more accurately. While for general news and business news, they are less able to accurately report preprint content.

In conclusion, legacy media performs less well in this area than digital native media when measured by the preprint frame adoption score. As we explore further, we can find that digital-native media have higher adoption scores because most medical and scientific content is published on digital platforms. To some extent, therefore, we can assume that media content influences preprint coverage quality more than media type.

## 5. Discussion and conclusion

Preprints have become an important source of information during the COVID-19 pandemic as the public demands quicker access to scientific information. This paper explores the current state of communication of preprints in the public sphere to help better understand the changing picture of science communication during the pandemic. In this way, we may be able to better respond to public health emergencies and maintain public confidence in the media and science.

The findings of this paper show that the COVID-19 pandemic has indeed increased the exposure of preprints in the public eye, suggesting the emergence of a new model of science communication. In previous models of science communication, scientists conducted research, published their findings in peer-reviewed journals through a long publication process, and then communicated their findings to the public in journalistic reports. This paper finds that the epidemic has changed this model, resulting in more scientific discoveries being disseminated into the public domain through preprinted papers. Several previous studies have shown that journalists have relied more on preprint as a source of news coverage due to the immediacy and ease of accessibility (33, 35). It is believed that journalists and the media are “knowledge brokers” who bridge the gap between science and society (53, 54). With the introduction of preprints into mainstream media under the pandemic, accuracy in reporting ongoing scientific research has implications for public trust in science and media, and science communication should therefore pay attention to this topic.

This study reveals that the overall media coverage of preprints is unsatisfactory, whereas digital native news media, particularly those specialized news media, possess higher reporting standards than legacy media. According to the study, only half of the news stories mentioned the preprint nature of the papers covered, and most failed to explain what that means. Comparing this result with previous similar studies, the proportion of stories that are not correctly reported is higher (42–44).

This study focuses on preprint papers that were not ultimately published in peer-reviewed journals. Previous research has primarily focused on all preprint papers, so it is likely that the papers in our sample have some qualitative differences in content compared to those that were included in previous studies. If a significant proportion of these preprint papers contain errors in their reports, it could have serious consequences. This underscores the need to improve the quality of these reports.

In addition, this paper found that digital native news media performed better than legacy media in reporting preprints accurately, which offers new ideas for reshaping the structure of science communication. Much of the previous literature on science communication coverage has focused on legacy or social media, with relatively little attention paid to digital native media (55–57). With science journalism declining in legacy media in recent years (58), the conclusions of this paper demonstrate the need to pay special attention to the role of digital native media in science communication, which appears to be a viable strategy for reshaping science communication (59, 60).

The findings of this study contribute to understanding how science communication has evolved in response to the COVID-19 pandemic. Firstly, in terms of the sources of science communication, it is essential to note that preprints are being widely disseminated to the general public. Secondly, it is necessary to improve the quality of reporting of preprints in the mass media. Finally, it is crucial to recognize the increasing importance of digital native media, especially when science journalism is declining in legacy media. Overall, this study indicates that public health emergencies significantly impact science communication.

Based on the findings, the following practical recommendations are made.

(1) Journalists should realize that preprints are not peer-reviewed and should not view them as the same papers in peer-reviewed journals. One way to make journalists aware of the nature of preprints would be to provide them with information and resources about preprints through training sessions.
(2) When journalists report on preprints, explain accordingly. A study has shown that even brief explanations can help non-scientists distinguish between preprints and peer-reviewed papers (61).
(3) When covering preprints, it is advisable to interview other researchers in the field to assist with assessing and explaining the significance of the preprint (62).
(4) Science communication should take full advantage of native digital media.

Science communication is challenged by the COVID-19 pandemic, and preprints serve as an effective tool to address this issue. Throughout this paper, we examine the current state of preprint communication in public view to better understand how science communication has evolved in the context of public health emergencies. Additionally, the paper provides practical suggestions for reporting preprints, which can help promote trust both in science and in the media.

Some limitations exist in this study. Firstly, the content analysis of the coverage on preprints focuses only on the English-language media and therefore lacks a broader global perspective. In addition, only reporting of preprints at the early stages of the pandemic was examined, ignoring the possibility that the reporting of preprints could change as the pandemic progresses. Therefore, future research could expand the scope of this study in terms of language and time. Finally, future research could examine changes in audiences' attitudes and behavior following exposure to preprint reports.

## Data availability statement

The original contributions presented in the study are included in the article/supplementary material, further inquiries can be directed to the corresponding author.

## Author contributions

The author confirms being the sole contributor of this work and has approved it for publication.

## Footnotes

^1^In this case, it would be more accurate to state that SARS-CoV-2 and HIV share a very small genetic sequence, but that this sequence is also found in many other organisms. It is not correct to say that SARS-CoV-2 is similar to HIV, as this could lead to misunderstandings and incorrect conclusions.

^2^According to the description of the dataset, the published data was collected in December 2020. It is likely that many papers on COVID-19 had already been published by the time of the count, given the rapid pace at which new research on the virus was being published during the early days of the pandemic. This is supported by studies that found the average time from submission to publication was 60 days (https://direct.mit.edu/qss/article/1/3/1056/96126/Pandemic-publishing-Medical-journals-strongly), 83.8 days (https://academic.oup.com/rev/article/30/3/231/6064166#312682880) with a median of 20 days (https://www.medrxiv.org/content/10.1101/2020.06.22.20137653v1). It is reasonable to assume that very few papers were still in the process of being published at the time of the count.

^3^The publication data provided by BioRxiv and medRxiv can be considered reliable, as the websites automatically add links to the published versions of papers within several weeks of journal publication (https://www.biorxiv.org/content/about-biorxiv). Additionally, the authors of the papers often update this information themselves, which helps to increase the citations of their work. As such, the information provided by these websites can be considered trustworthy.

^4^I have decided to use 3 as the threshold because papers with three or more news items are more likely to be influential in the public eye. This is because such papers have a greater potential for dissemination to popular news sites, whereas papers with one or two news items are more likely to originate from public relations news from the organizations they belong to, or from a narrow range of specialist news sites.

^5^LexisNexis is a prominent company that offers online information services, including access to legal, news, and business-related data. This company's products and services are utilized by professionals such as lawyers, journalists, researchers, and others to search through a vast collection of legal, news, and business information. LexisNexis provides a range of tools and resources that are designed to help users quickly and easily find the information they need.

^6^In this paper, we count tweets with more than two mentions because preprint platforms typically post tweets of platform papers automatically. To reduce this effect, we count tweets with more than two mentions.

## References

[1] Bavel JJV Baicker K Boggio PS Capraro V Cichocka A Cikara M. Using social and behavioural science to support COVID-19 pandemic response. Nat Hum Behavi. (2020) 4:460–71. 10.1038/s41562-020-0884-z 32355299

[2] Battiston P Kashyap R Rotondi V. Reliance on scientists and experts during an epidemic: evidence from the COVID-19 outbreak in Italy. SSM Popul Health. (2021) 13:100721. 10.1016/j.ssmph.2020.100721 33553567 PMC7859315

[3] Huisman J Smits J. Duration and quality of the peer review process: the author's perspective. Scientometrics. (2017) 113:633–50. 10.1007/s11192-017-2310-5 29056794 PMC5629227

[4] Xing W Hejblum G Leung GM Valleron AJ. Anatomy of the epidemiological literature on the 2003 SARS outbreaks in Hong Kong and Toronto: a time-stratified review. PLoS Med. (2010) 7:e1000272. 10.1371/journal.pmed.1000272 20454570 PMC2864302

[5] Sarabipour S Debat HJ Emmott E Burgess SJ Schwessinger B Hensel Z. On the value of preprints: an early career researcher perspective. PLoS Biol. (2019) 17:e3000151. 10.1371/journal.pbio.3000151 30789895 PMC6400415

[6] Wang Z Chen Y Glänzel W. Preprints as accelerator of scholarly communication: an empirical analysis in Mathematics. J Informetr. (2020) 14:101097. 10.1016/j.joi.2020.101097

[7] Puebla I Polka J and Rieger O. Preprints: their evolving role in science communication *MetaArXiv Preprints*. (2021). 10.31222/osf.io/ezfsk

[8] Berg JM Bhalla N Bourne PE Chalfie M Drubin DG Fraser JS. Preprints for the life sciences. Science. (2016) 352:899–901. 10.1126/science.aaf9133 27199406

[9] Wang Z Glänzel W Chen Y. How self-archiving influences the citation impact of a paper. In: Proceedings of the 23rd International Conference on Science and Technology Indicators. Leiden: CWTS, Leiden University (2018). p. 323–30.

[10] Gianola S Jesus TS Bargeri S Castellini G. Characteristics of academic publications, preprints, and registered clinical trials on the COVID-19 pandemic. PLoS ONE. (2020) 15:e0240123. 10.1371/journal.pone.0240123 33022014 PMC7537872

[11] Jung YEG Sun Y Schluger NW. Effect and reach of medical articles posted on preprint servers during the COVID-19 pandemic. JAMA Intern Med. (2021) 181:395–7. 10.1001/jamainternmed.2020.6629 33165545 PMC7653533

[12] Majumder MS Mandl KD. Early in the epidemic: impact of preprints on global discourse about COVID-19 transmissibility. Lancet Glob Health. (2020) 8:e627–30. 10.1016/S2214-109X(20)30113-3 32220289 PMC7159059

[13] Koerber A. Is it fake news or is it Open Science? Science communication in the COVID-19 pandemic. J Business Tech Commun. (2021) 35:22–7. 10.1177/1050651920958506

[14] Pradhan P Pandey AK Mishra A Gupta P Tripathi PK Menon MB. Uncanny similarity of unique inserts in the 2019-nCoV spike protein to HIV-1 gp120 and Gag. bioRxiv. (2020). 10.1101/2020.01.30.927871

[15] Pellechia MG. Trends in science coverage: a content analysis of three US newspapers. Public Understand Sci. (1997) 6:49. 10.1088/0963-6625/6/1/004

[16] Vestergård GL Nielsen KH. From the preserves of the educated elite to virtually everywhere: a content analysis of Danish science news in 1999 and 2012. Public Understand Sci. (2017) 26:220–34. 10.1177/0963662515603272 26386021

[17] Elmer C Badenschier F Wormer H. Science for everybody? How the coverage of research issues in German newspapers has increased dramatically. Journalism Mass Commun Quart. (2008) 85:878–93. 10.1177/107769900808500410

[18] Suleski J Ibaraki M. Scientists are talking, but mostly to each other: a quantitative analysis of research represented in mass media. Public Understand Sci. (2010) 19:115–25. 10.1177/0963662508096776

[19] Clark F Illman DL. A longitudinal study of the New York Times Science Times section. Sci Commun. (2006) 27:496–513. 10.1177/1075547006288010

[20] Weitkamp E. British newspapers privilege health and medicine topics over other science news. Public Relat Rev. (2003) 29:321–33. 10.1016/S0363-8111(03)00041-9

[21] Phillips DP Kanter EJ Bednarczyk B Tastad PL. Importance of the lay press in the transmission of medical knowledge to the scientific community. N Engl J Med. (1991) 325:1180–3. 10.1056/NEJM199110173251620 1891034

[22] McKiernan EC Bourne PE Brown CT Buck S Kenall A Lin J. How open science helps researchers succeed. Elife. (2016) 5. 10.7554/eLife.16800.008 27387362 PMC4973366

[23] Vestergård GL. From journal to headline: the accuracy of climate science news in Danish high quality newspapers. J Sci Commun. (2011) 10:A03. 10.22323/2.10020203

[24] Tankard Jr JW Ryan M. News source perceptions of accuracy of science coverage. Journalism Quart. (1974) 51:219–25. 10.1177/107769907405100204

[25] Matthias L Fleerackers A Alperin JP. Framing science: how opioid research is presented in online news media. Front Commun. (2020) 5:64. 10.3389/fcomm.2020.00064

[26] Sumner P Vivian-Griffiths S Boivin J Williams A Venetis CA Davies A. The association between exaggeration in health related science news and academic press releases: retrospective observational study. BMJ. (2014) 349:g7015. 10.1136/bmj.g7015 25498121 PMC4262123

[27] Adams RC Challenger A Bratton L Boivin J Bott L Powell G. Claims of causality in health news: a randomised trial. BMC Med. (2019) 17:91. 10.1186/s12916-019-1324-7 31092248 PMC6521363

[28] Molitor F. Accuracy in science news reporting by newspapers: the case of aspirin for the prevention of heart attacks. Health Commun. (1993) 5:209–24. 10.1207/s15327027hc0503_4

[29] Brechman JM Lee CJ Cappella JN. Distorting genetic research about cancer: from bench science to press release to published news. J Commun. (2011) 61:496–513. 10.1111/j.1460-2466.2011.01550.x 25580022 PMC4287246

[30] da Silva JAT. Adjusting the use of preprints to accommodate the “quality” factor in response to COVID-19. J Taibah Univ Med Sci. (2021) 16:477–81. 10.1016/j.jtumed.2021.04.003 34408603 PMC8348262

[31] Johansson MA Reich NG Meyers LA Lipsitch M. Preprints: an underutilized mechanism to accelerate outbreak science. PLoS Med. (2018) 15:e1002549. 10.1371/journal.pmed.1002549 29614073 PMC5882117

[32] Fraser N Brierley L Dey G Polka JK Pálfy M Nanni F. The evolving role of preprints in the dissemination of COVID-19 research and their impact on the science communication landscape. PLoS Biol. (2021) 19:e3000959 10.1371/journal.pbio.3000959 33798194 PMC8046348

[33] Makri A. What do journalists say about covering science during the COVID-19 pandemic? Nat Med. (2021) 27:17–20. 10.1038/s41591-020-01207-3 33442019

[34] Massarani L Luiz FN Entradas M Lougheed T Bauer MW. Perceptions of the impact of the COVID-19 pandemic on the work of science journalists: global perspectives. J Sci Commun. (2021) 20. 10.22323/2.20070206

[35] Fleerackers A Moorhead L Maggio LA Fagan K Alperin JP. Science in motion: a qualitative analysis of journalists' use and perception of preprints. Plos One. (2022) 17:e0277769. 10.1371/journal.pone.0277769 36409723 PMC9678308

[36] Besançon Besançon L Peiffer-Smadja N Segalas C Jiang H Masuzzo P Smout C. Open science saves lives: lessons from the COVID-19 pandemic. BMC Med Res Methodol. (2021) 21:117. 10.1186/s12874-021-01304-y 34090351 PMC8179078

[37] Waters AM LeBeau RT Young KS Dowell TL Ryan KM. Towards the enhancement of quality publication practices in clinical psychological science. Behav Res Ther. (2020) 124:103499. 10.1016/j.brat.2019.103499 31751896

[38] Sheldon T. Preprints could promote confusion and distortion. Nature. (2018) 559:445–6. 10.1038/d41586-018-05789-4 30042547

[39] Donahue MZ. An avid new audience for preprints—extremists: preprints can speed scientific communication but offer fuel for nefarious agendas. Bioscience. (2021) 71:1004–10. 10.1093/biosci/biab090

[40] Sizo A Lino A Reis LP Rocha Á. An overview of assessing the quality of peer review reports of scientific articles. Int J Inf Manag. (2019) 46:286–93. 10.1016/j.ijinfomgt.2018.07.00234647763

[41] Fleerackers A Riedlinger M Moorhead L Ahmed R Alperin JP. Communicating scientific uncertainty in an age of COVID-19: an investigation into the use of preprints by digital media outlets. Health Commun. (2022) 37:726–38. 10.1080/10410236.2020.1864892 33390033

[42] van Schalkwyk F Dudek J. Reporting preprints in the media during the COVID-19 pandemic. Public Understand Sci. (2022) 31:608–16. 10.1177/09636625221077392 35196912 PMC9160779

[43] Oliveira T Araujo RF Cerqueira RC Pedri P. Politização de controvérsias científicas pela mídia brasileira em tempos de pandemia: a circulação de preprints sobre COVID-19 e seus reflexos. Rev Brasil Hist Mí*dia*. (2021) 10. 10.26664/issn.2238-5126.101202111810

[44] Massarani L Neves LFF. Reporting COVID-19 preprints: fast science in newspapers in the United States, the United Kingdom and Brazil. Ciência Saúde Coletiva. (2022) 27:957–68. 10.1590/1413-81232022273.20512021 35293473

[45] Else H. How a torrent of COVID science changed research publishing–in seven charts. Nature. (2020) 588:553–4. 10.1038/d41586-020-03564-y 33328621

[46] Strcic J Civljak A Glozinic T Pacheco RL Brkovic T Puljak L. Open data and data sharing in articles about COVID-19 published in preprint servers medRxiv and bioRxiv. Scientometrics. (2022) 127:2791–802. 10.1007/s11192-022-04346-1 35370324 PMC8956135

[47] DuPre NC Karimi S Zhang CH Blair L Gupta A Alharbi LMA. County-level demographic, social, economic, and lifestyle correlates of COVID-19 infection and death trajectories during the first wave of the pandemic in the United States. Sci Total Environ. (2021) 786:147495. 10.1016/j.scitotenv.2021.147495 33971599 PMC8091799

[48] Davillas A Jones AM. The first wave of the COVID-19 pandemic and its impact on socioeconomic inequality in psychological distress in the UK. Health Econ. (2021) 30:1668–83. 10.1002/hec.4275 33904203 PMC8207020

[49] Iftimie F López-Azcona AF Vallverdú I Hernàndez-Flix S de Febrer G Parra S. second waves of coronavirus disease-19: a comparative study in hospitalized patients in Reus, Spain. PLoS One. (2021) 16:e0248029. 10.1371/journal.pone.0248029 33788866 PMC8011765

[50] Gaudart J Landier J Huiart L Legendre E Lehot L Bendiane MK. Factors associated with the spatial heterogeneity of the first wave of COVID-19 in France: a nationwide geo-epidemiological study. Lancet Public Health. (2021) 6:e222–31. 10.1016/S2468-2667(21)00006-2 33556327 PMC7864788

[51] Fraser N Brierley L Dey G Polka J Palfy M Nanni F Coates J. Preprinting-a-pandemic/pandemic_preprints: Updated analysis. Zenodo (2022). Available online at: https://zenodo.org/record/4501924#.YuIKkOxBy3K (accessed July 18, 2022).

[52] About Us. Altmetric. (2019). Available online at: from https://www.altmetric.com/about-us/ (accessed August 25, 2022).

[53] Rajkhowa A. COVID-19 dissensus in Australia: negotiating uncertainty in public health communication and media commentary on a pandemic. Pacific Journalism Rev. (2020) 26:253–63. 10.24135/pjr.v26i1.1091

[54] Pentzold C Fechner DJ Zuber C. “Flatten the Curve”: data-driven projections and the journalistic brokering of knowledge during the COVID-19 crisis. Digit Journalism. (2021) 9:1367–90. 10.1080/21670811.2021.1950018

[55] Oh SH Lee SY Han C. The effects of social media use on preventive behaviors during infectious disease outbreaks: the mediating role of self-relevant emotions and public risk perception. Health Commun. (2021) 36:972–81. 10.1080/10410236.2020.1724639 32064932

[56] Chan MPS Winneg K Hawkins L Farhadloo M Jamieson KH Albarracín D. Legacy and social media respectively influence risk perceptions and protective behaviors during emerging health threats: a multi-wave analysis of communications on Zika virus cases. Soc Sci Med. (2018) 212:50–9. 10.1016/j.socscimed.2018.07.007 30005224 PMC6093206

[57] Schäfer MS. How changing media structures are affecting science news coverage. Oxford Handb Sci Sci Commun. (2017) 51:57. 10.1093/oxfordhb/9780190497620.013.5

[58] Friedman SM. The changing face of environmental journalism in the United States. In: The Routledge Handbook of Environment and Communication. Routledge (2015). p. 164–226.

[59] Painter J Kristiansen S Schäfer MS. How ‘digital-born'media cover climate change in comparison to legacy media: a case study of the COP 21 summit in Paris. Glob Environ Change. (2018) 48:1–10. 10.1016/j.gloenvcha.2017.11.003

[60] Díaz-Pont J Egan Sjölander A Foxwell-Norton K Mishra M Maeseele P. Environmental Communication in the Intertwining of the Local and the Digital. In: The Local and the Digital in Environmental Communication. Cham: Palgrave Macmillan (2020). p. 1–29. 10.1007/978-3-030-37330-6_1

[61] Wingen T Berkessel JB Dohle S. Caution, preprint! Brief explanations allow nonscientists to differentiate between preprints and peer-reviewed journal articles. Adv Methods Pract Psychol Sci. (2022) 5:25152459211070559. 10.1177/25152459211070559

[62] Ordway D-M. Covering research preprints amid the coronavirus: 6 things to know. The Journalist's Resource (2022). Available online at: https://journalistsresource.org/health/medical-research-preprints-coronavirus/ (accessed August 25, 2022).

